# Effect of Acid and Base Catalyzed Hydrolysis on the Yield of Phenolics and Antioxidant Activity of Extracts from Germinated Brown Rice (GBR)

**DOI:** 10.3390/molecules17067584

**Published:** 2012-06-19

**Authors:** Ismaila Muhammad Sani, Shahid Iqbal, Kim Wei Chan, Maznah Ismail

**Affiliations:** 1 Laboratory of Molecular Biomedicine, Institute of Bioscience, UniversitiPutra Malaysia, Serdang 43400, Selangor, Malaysia; 2 Department of Pharmacology and Toxicology, Faculty of Veterinary Medicine, Usmanu Danfodiyo University, Sokoto 2346, Nigeria; 3 Department of Chemistry, University of Sargodha, Sargodha 40100, Pakistan; 4 Department of Nutrition and Dietetics, Faculty of Medicine and Health Science, Universiti Putra Malaysia, Serdang 43400, Selangor, Malaysia

**Keywords:** antioxidant activity, germinated brown rice, hydrolysis, HPLC, phenolics, yield

## Abstract

The influence of both acidic and basic hydrolysis on the yield, total phenolic content and antioxidative capacity of methanolic extract of germinated brown rice (GBR) was studied. Total phenolic content (TPC), total flavonoid content (TFC), 2,2′-diphenyl-1-picrylhydrazyl (DPPH) radical scavenging, 2,2′-azino-bis(3-ethylbenzothiazoline-6-sulphonic acid (ABTS) radical cation scavenging, and ferric reducing antioxidant power (FRAP) tests were used for the measurement of antioxidant ability. There was a significant difference (*p* < 0.05) in the TPC and DPPH radical scavenging assay results when comparing neutral with acidic and basic catalysed hydrolysis. The yield of the crude extract was slightly higher in acidic hydrolysis than in basic hydrolysis (*p* > 0.05). The TPC and TFC were highest in acidic hydrolysis. A significant correlation was observed between ABTS radical cation scavenging and FRAP. The antioxidant activity measured using DPPH radical scavenging assay showed high activity in acidic hydrolysis, while the ABTS radical cationscavenging activity and FRAP showed the highest values in basic hydrolysis. The samples were further evaluated using HPLC to determine the individual phenolic concentrations in different hydrolytic media contributing to the antioxidant effects. This study revealed that acidic and basic hydrolysis can improve the yield, phenolic content, and antioxidant activity of germinated brown rice.

## 1. Introduction

Over the last decade, germinated brown rice (GBR) has attracted much attention as a nutraceutical health-promoting agent. Besides its applications in nutraceuticals and functional food research, considerable research has been conducted on its role in disease prevention as well as management. It has been shown that brown rice has more nutritional components than white rice. These components include phytic acid, dietary fibre, vitamin E, vitamin B, and γ-aminobutyric acid (GABA), most of which occur mainly in the outer layer and are lost during polishing [1]. A number of studies have been presented demonstrating that the germination process may significantly enhance the content of nutritional and disease-preventing phenolics and also improve the antioxidant effects of GBR [1,2,3,4]. Cereals are generally known for their content of phenolic compounds, which have the capability of suppressing oxidative stress, which in turn is responsible for a number of metabolic diseases; most of these properties are linked to their intrinsic reducing ability [5]. Phenolics are reported to have the ability to neutralise free radicals by quenching unpaired oxygen radicals [6]. The ability of antioxidants in GBR to ameliorate some disease pathologies caused by oxidative stress, such as cancer and diabetes, has encouraged scientists to focus their research on the exploration of these antioxidants and their possible applications in disease management. Ryan *et al.* [7] reported that the extraction of phenolic compounds from natural substances is complicated by their diversity and sensitivity to oxidation and hydrolysis, which are often neglected. Despite the fact that quite a number of studies have examined the phenolic and antioxidant contents of various rice species, rice bran and germinated brown rice [8,9,10,11], effort has not been focused on the effect of acidic and basic hydrolysis on the yield and antioxidant activity of GBR. Therefore, this study, aimed to explore the effect of acidic and basic hydrolysis on the yield and antioxidant effects of GBR was conducted by the application of various antioxidant assays, and further to elucidate the concentration of the individual phenolics in different catalytic reactions.

## 2. Results and Discussion

### 2.1. Yield of the GBR Crude Extract

**Figure 1:**
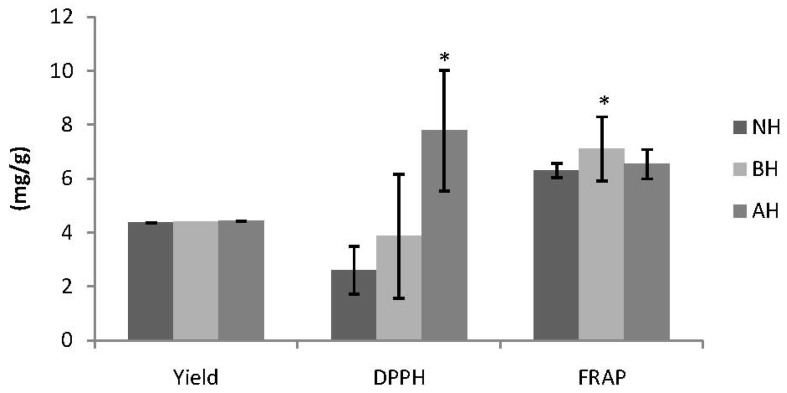
Total yield of crude extract, DPPH radical scavenging activity, and FRAP assays following acidic and basic catalysed hydrolysis of germinated brown rice (GBR).

Methanol is usually recommended for the extraction of antioxidant compounds [12]. The effectiveness of methanol has been shown to enhance the extraction of bioactives from cereals such as rice when water is added as the co-solvent, especially in the extraction of a complex mixture of antioxidant compounds [13]. Previous studies have reported high antioxidant activities from methanolic extracts of grains compared to other solvents [14]. The yield of the crude extract is shown in Figure 1. The yield was slightly higher in acidic hydrolysed medium than in the basic medium, although this was not significant (*p* > 0.05). This showed that acidic and basic hydrolysis can enhance the yield of extraction in germinated brown rice, and the enhancement is facilitated in both acidic and the basic media. High yield and antioxidant activity was obtained in acidic hydrolysates in the solvent extraction of almond shells [15]. It has also been reported [16] that 80% methanol in acidic hydrolysates increases the yield of quercetin in idared apple peals. It has been shown that the hydrolysis method can improve the yield and antioxidant capability of wheat [17,18]. These results also show that there was a good correlation between TPC, TFC, DPPH radical scavenging, and the yield of GBR.

### 2.2. Total Phenolic Content

**Figure 2:**
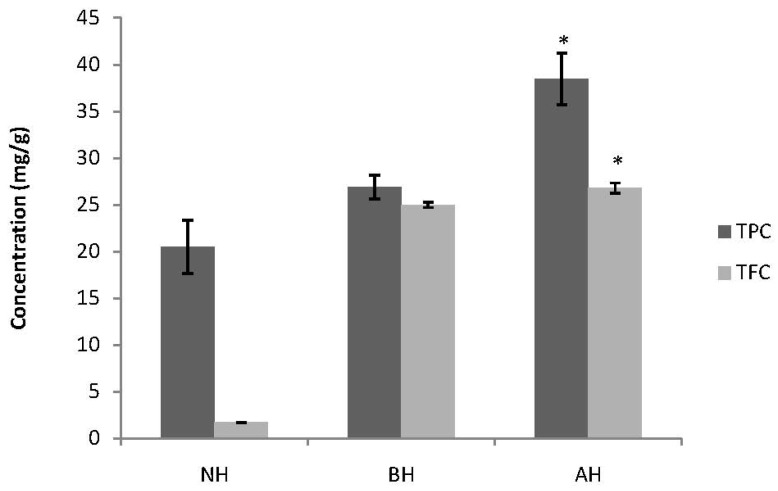
Total phenolic content (TPC) and total flavonoid content (TFC) following acidic and basic catalyzed hydrolysis in germinated brown rice (GBR).

Hydrolysis enhanced the total phenolic content in this study, and as with the yield, acidic hydrolysis gave the highest concentration of phenolics (Figure 2). Phenolics are generally known to exist in two forms: Free and bound. Bound phenolics can be hydrolysed using an acid or a base; our study shows that bound phenolics in GBR were released or extracted more readily in acidic hydrolysis. Our study results coincide with earlier researchers [14], who reported the same in wheat bran. They further revealed that the major phenolic acids in wheat were not extractable by aqueous methanol, but were released only upon alkaline or acid hydrolysis. Phenolics in wheat occur mostly in the bound form, and that the amount of bound phenolics is 2.5 to 5.4 fold higher than the free phenolic content in wheat grain [19]. It has been reported that brown rice contains about three times more polyphenolics compared to white rice [14]. TPC of different rice varieties, including GBR were compared [10], but studies on free and bound phenolics in rice are lacking. Our results show significant differences (*p* < 0.05) when comparing the TPC between the results of the three different groups, and there was a correlation between DPPH radical scavenging, TFC and TPC in this study (R^2^ = 0.811 and 0.992, respectively). This may be attributed to acidic and basic hydrolysis, which extracted more of the bound and soluble phenolic compounds.

### 2.3. Total Flavonoid Content

Flavonoids are the most abundant polyphenols in our diets. There was a significant increase in TFC after hydrolysis, as shown in Figure 2. TFC values were higher in acidic hydrolysis than in neutral or basic hydrolysis. There was a correlation between DPPH radical, ABTS radical cation, and FRAP scavenging activity with their respective total yield and the TFC (R^2^ = 0.733, 0.704 and 0.679, respectively), but there was no significant difference between the three groups (*p* < 0.05). This suggests that polyphenols play an important role in the antioxidant activity of germinated brown rice and they may be extracted more readily when hydrolysis is employed.

### 2.4. DPPH Radical Scavenging Activity

The measurement of scavenging activity of the 2,2′-diphenyl-1-picrylhydrazyl (DPPH) radical by spectrophotometry is one of the most frequently used assays in the determination of antioxidant activity in natural products. The scavenging activity was higher in acidic than in basic hydrolysis, as shown in Figure 1. This shows that the scavenging activity, measured using the DPPH radical, was higher in acidic hydrolysis. The study also concurs with a previous report in wheat; where [20] the highest DPPH radical scavenging activity was reported in the acid labile fraction of a particular wheat preparation. There was a correlation between DPPH and TPC as well as TFC. To the best of our knowledge, no other study has been conducted on the DPPH radical scavenging activity of GBR using acidic and basic hydrolysis.

### 2.5. ABTS Radical Cation Scavenging Activity

**Figure 3:**
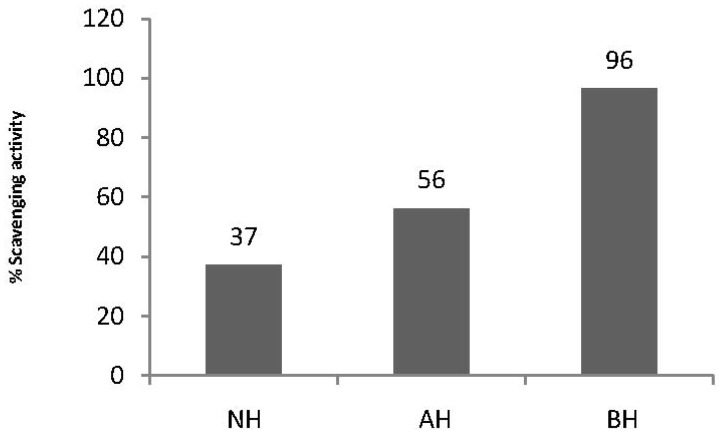
ABTS Radical Cation Scavenging Activity of GBR following acidic and basic hydrolysis.

ABTS is an important method for the determination of radical scavenging activity in plant materials and grains. Our results showed higher scavenging activity in basic than in acidic hydrolysis (Figure 3). Our ABTS results correlate well with our FRAP assay results (*p* < 0.05). Other workers also reported a high correlation between ABTS and FRAP assays [21]. A correlation was also observed between ABTS and DPPH (R^2^ = 0.733). The same correlation was reported between the two assays [22] in sorghum. This may be due to fact that the two assays worked by the same principle and mechanism.

### 2.6. FRAP Assay

Although this test was developed initially to assay plasma antioxidant capacity, it has also been widely used in the determination of antioxidant capacity in wide range of pure compounds and biological samples [23]. The test works on the principles of electron donation by an antioxidant. It measures changes in absorbance by the formation of blue iron (II) from colourless oxide of iron (III). In our results, the FRAP assay showed that basic hydrolysis had a high ferric reducing antioxidant capacity (Figure 1). This clearly shows that hydrolysis affects the antioxidant capability of GBR. We also observed a positive and significant correlation between the ABTS radical cation scavenging activity and FRAP results (R^2^ = 0.999 and *p* = 0.022). A correlation observed between FRAP and TFC was also reported [12], owing to the fact that flavonoids can act as antioxidants by acting as hydrogen donors or as chelating agents.

### 2.7. HPLC of Phenolic Compounds

Table 1 shows the standard calibration curves of standards (area *vs*. concentration) showingretention time, area, as well as concentrations of various phenolic acid standards, which were run in triplicates together with the samples using the Agilent HPLC instrument. The basic fraction contained more phenolics than the acidic or neutral media in this study, as shown in Table 2. Gallic, *p*-coumaric, vanillic and ferulic acids were the phenolic acids expressed in the acidic catalysed fraction, while in the basic hydrolysed fraction, gallic, hydrocinnamic, caffeic, syringic, protocatechuic, as well as *p*-coumaric acids were recorded. It has been previously reported that *p*-coumaricand ferulic acids are the major phenolic acids expressed in rice [24]. The disappearance of some of the phenolics in the acidic medium may have been due to their destruction during acidic hydrolysis, as reported earlier in wheat [18]. Our results indicate that *p*-coumaricand ferulic acids, which are the major phenolics in rice, were expressed in acidic medium, while only *p*-coumaric acid was expressed in basic medium, although this component had the highest expression in terms of individual phenolics (Table 2). This might be a reason why some of the antioxidant assays gave good results in acidic medium, while others gave the best results in basic medium.

## 3. Experimental

### 3.1. Materials

Brown rice samples were collected from the PadiBeras National (Barnas) rice company Malaysia. Samples were subjected to germination according to the pre-optimised conditions established in our laboratory [as mentioned in 3.1.2].

#### 3.1.1. Reagents and Chemicals

Ferricchloride, potassiumferricyanide, rutin, potassium persulphate, 2,2-azino-bis(3-ethylbenzo-thiazoline-6-6-sulphonic acid), gallic acid, 1,1-diphenyl-2-picrylhydrazyl (DPPH), Trolox, aluminiumtrichloride, sodium carbonate, hydrochloric acid, and phenolic standards were procured from Sigma Aldrich (Hamburg, Germany). Folin-Ciocalteau reagent and sodium hydroxide were purchased from Merck (Damastadt, Germany). Methanol, acetic acid, acetonitrile, and phosphoric acid used in HPLC analysis were purchased from Fisher Scientific (Loughborough, Leicestershire, UK). All the reagents and solvents were of analytical or HPLC grade.

#### 3.1.2. Preparation of GBR

Twenty grams of brown rice was measured; the moisture content was found to be 8.34%. The rice was then washed twice using clean tap water and drained for 5minutes, then soaked in sodium hypochlorite for 30 min at a ratio of 1:2 (w/v), then drained again and rinsed. The moisture content was determined before soaking the rice in 1:2 (w/v) H_2_O_2_ at 37 °C at a final concentration of 0.5% for 6 h, then it was incubated for 18 h in a triangular plastic container at 37 °C. The percentage of germination was calculated by taking 10% of the total GBR for counting. The material was finally dried in an oven at 50 °C. The final moisture content was 8.43%. The GBR was then cooled before transfer into zipped nylon bags and stored at 4 °C.

#### 3.1.3. Extraction

Germinated brown rice (GBR, 15 g) was ground into a powder and then divided into three parts containing 5 g in each of three conical flasks; the material was then subjected to extraction using 80% methanol (50 mL) for 2 h in an electric shaker (extraction was performed three times). The first sample was non-hydrolysed and was designated as (NHS); 1 M HCl (200 µL) was added to the second sample, which was designated as the acidic hydrolysed sample (AHS); 1M NaOH was added to the third sample, which was designated as the basic hydrolysed sample (BHS). The extracts were filtered and then evaporated at 50 °C at a pressure of 206 mmHg using a Buchi R-210 rotary evaporator (Rotavap^®^), then freeze dried at −80 °C in a freeze drier and stored at 4 °C before analysis.

#### 3.1.4. Total Phenolic Content (TPC)

The total phenolic content was determinedby the Folin-Ciocalteau method. Precisely, 200 µL of the extract was mixed with freshly prepared 1:10 Folin-Ciocalteau reagent (800 µL) in a test tube, then 20% Na_2_CO_3_ (2 mL) was added to the mixture and the three samples were stored in the dark for 2 h to complete the reaction. The samples and the standard (gallic acid) were prepared in triplicate. The absorbance of the samples andthe standard was then measured at 760 nm using a spectrophotometer, and the results were expressedasmg GAE/g d.w. sample.

#### 3.1.5. Total Flavonoid Content (TFC)

To determine the total flavonoid content, the sample extract (100 µL) was reacted with 2% AlCl_3_ (100 µL in a 96-well plate, and incubated at room temperature for 10 min. Rutin was used as the standard at a concentration of 1 mg/mL of methanol. AlCl_3_ was mixed with methanol, serving as the control (blank). The standards, the blank, and the samples were prepared in triplicate and the absorbance was measured at 405 nm using a microplate reader.

#### 3.1.6. DPPH Radical Scavenging Activity

DPPH radical scavenging activity was measured according to a standard procedure described earlier [25] with a slight modifications. Briefly, a 1000 ppm test sample was prepared by dissolving GBR (1 mg) in methanol (1 mL). Trolox was used as the standard. The test sample containing 50 µL was mixed with 0.1 mM DPPH radical methanolic solution (195 µL) in a 96-well microplate. The plate contents were then gently mixed for about 1 min, and the absorbance was measured after the sample was left in dark for 60 min. The sample and the standard were prepared in triplicate. A standard graph was then plotted based on the absorbance and the results were recorded in mg TE/g d.w. sample.

#### 3.1.7. ABTS Radical Cation Scavenging Activity

This was estimated according to the method described earlier [26]. In brief, 2.45 mM potassium persulphate was added to 7 mM ABTS to make a stock solution, which was left in the dark at room temperature for 16 h. The resulting ABTS radical cationwas then dissolved in methanol to give an absorbance of 0.7 ± 0.02 at 734 nm. ABTS radical cation (900 µL) was added to the test sample (100 µL) or Trolox, which was used as the standard. Methanol served as the blank, then ABTS radical cationwith methanol as the control. The absorbance was measured at 734 nm. ABTS radical cation scavenging activity was expressed as mg TE/g d.w. sample.

#### 3.1.8. FRAP Assay

The ferric reducing antioxidant power of the extracts was determined in this study by reacting the extract (1 mL) with distilled water (5 mL), then adding 1 M HCl (1.5 mL) and 1% potassium ferricyanide (1.5 mL). Then sodium dodecyl sulphate (SDS, 0.5 mL) and 0.5 mL of 0.2% ferric chloride (FeCl_3_) were added, and the entire solution was incubated at 50 °C for 20min to complete the reaction. Gallic acid was used as the standard and the absorbance was measured using a spectrophotometer at 750 nm, and the results were expressed as mg GAE/g d.w. sample.

#### 3.1.9. HPLC Analysis

HPLC analysis was performed using Agilent G1310A pumps (Agilent, Santa Clara, CA, USA) with a diode array detector set at wavelengths of 280 nm and 320 nm. Chromatographic separations were performed on a LUNA C-18 column (5 mm, 250 × 4.6 mm) (Phenomenex, Torrance, CA, USA). The solvent composition and gradient elution conditions used were the same as those described previously [27]. The mobile phase was composed of solvent (A) water–acetic acid (94:6, v/v, pH 2.27) and solvent (B) acetonitrile. The solvent gradient was as follows: 0–15% B for 40 min, 15–45% B for40 min, and 45–100% B for10 min. A flow rate of 0.5 mL/min was used and 20 μL of sample were injected. Samples and mobile phases were filtered through a 0.22 μm Millipore filter, type GV (Millipore, Bedford, MA, USA) prior to HPLC injection. Each fraction was analysed in triplicate. Determination and quantification of phenolic compounds were done by comparing their retention times and UV–Vis spectral data to known, previously injected standards.

#### 3.1.10. Statistical Analysis

All the experiments were conducted in triplicate and the data are expressed as mean ± SD. Differences within the three media were compared by analysis of variance (ANOVA); mean comparisons for all the pairs were performed using the Tukey-Kramer HSD test. The results of the phenolic content assay were correlated with those of the various antioxidant tests. Data were considered significant at *p* < 0.05 using JMP 9.0 Statistical Software and MINITAB 14.

## 4. Conclusions

The results of this study clearly show that acidic and basic hydrolysis has a significant impact on the yield, total phenolic content, and antioxidant activities of Malaysian germinated brown rice. The release of bound phenolic compounds may be enhanced by either acidic or basic hydrolysis; the choice of the medium depends on the individual phenolic compound of interest.

## Figures and Tables

Each value in the diagram represents a mean ± SD (n = 3); * Indicates a significant difference between the three different media (*p* < 0.05). NH = No hydrolysis, BH = Basic hydrolysis, AH = Acidic hydrolysis. Yield was expressed in mg/g, while DPPH radical scavenging activity in milligram Trolox equivalents per gram dry weight of sample (mg TE/g d.w.) and FRAP in milligram of gallic acid equivalents per gram dry weight of sample (mg GAE/g d.w.).

Each value in the diagram represent a mean ± SD (n = 3); * Indicates a significant difference between the three different media (*p* < 0.05). NH = No hydrolysis, BH = Basic hydrolysis, AH = Acidic hydrolysis. TPC was expressed in mg GAE/g d.w. of sample and TFC was expressed in mg RAE/g d.w. of sample.

NH = No hydrolysis, AH = Acidic hydrolysis, BH = Basic hydrolysis. Basic hydrolysis showed higher ABTS percentage scavenging activity with 96% *p* < 0.05.

**Table 1:** Linearity of standard calibration curves for various phenolic acid standards as determined by HPLC-UV.

Phenolic acid	Calibration curve range (µg/mL)	Regression equation	R^2^	Retention time (min)	Area (y) (mAU*s)	Concentration (x, µg/g)
Gallic	1.56–25	y= 57.342x − 0.3129	R^2^ = 1	2.267	39,544.9	689.64
Hydroxycinnamic	1.56–25	y = 33.738x − 2.0438	R^2^ = 1	6.256	517,169.8	15329
Chlorogenic	3.13–25	y = 4.4916x − 0.3016	R^2^ = 1	6.914	433,190.5	9644.45
Caffeic	1.56–25	y = 18.791x − 0.728	R^2^ = 1	8.387	19,381.3	1031.45
Vanillic	1.56–100	y = 36.364x − 0.3407	R^2^ = 1	8.567	40,229.6	1106.31
Syringic	1.56–100	y = 58.995x − 3.4366	R^2^ = 1	9.496	61,044.9	1038.81
Protocatechuic	6.25–50	y = 57.727x − 8.409	R^2^ = 0.9999	11.848	858,296.3	1486.83
*p*-Coumaric	1.56–100	y = 58.331x − 4.9255	R^2^ = 1	12.055	54,838.8	940.22
Ferulic	1.56–12.5	y = 13.942x − 0.6618	R^2^ = 0.9999	13.207	20,491.5	1469.82

Where y = Area (mAU*s), x = Concentration (µg/mL), R^2^ is the regression coefficient and the calibration curve range is in (µg/g), respectively.

**Table 2:** Retention time (min) and concentrations of phenolics following acidic and basic catalysedhy drolysis of germinated brown rice (GBR) as detected by HPLC-UV.

Retention Time (min)				Concentrations (µg/g)		
Phenolics	NH	AH	BH	NH	AH	BH
Gallic	ND	2.345 ± 0.06	2.311 ± 0.008	ND	9.516 ± 0.412	13.822 ± 1.88
Hydroxycinnamic	ND	ND	6.222 ± 0.014	ND	ND	17.465 ± 0.11
Chlorogenic	ND	ND	ND		ND	ND
Caffeic	ND	ND	8.394 ± 0.008	ND	ND	61.319 ± 0.12
Vanillic	ND	8.603 ± 0.07	ND	ND	3.319 ± 0.53	ND
Syringic	ND	ND	9.584 ± 0.009	ND	ND	10.499 ± 0.02
Protocatechuic	ND	ND	11.876 ± 0.009	ND	ND	13.816 ± 0.12
*p*-Coumaric	12.140 ± 0.014	12.105 ± 0.098	12.116 ± 0.004	8.670 ± 1.32	5.567 ± 0.06	9.122 ± 0.94
Ferulic	13.179 ± 0.012	13.202 ± 0.013	ND	7.96 ± 0.662	13.212 ± 0.82	ND

ND = not detected.
